# ATX-LPA-Dependent Nuclear Translocation of Endonuclease G in Respiratory Epithelial Cells: A New Mode Action for DNA Damage Induced by Crystalline Silica Particles

**DOI:** 10.3390/cancers15030865

**Published:** 2023-01-30

**Authors:** Huiyuan Zheng, Ulla Stenius, Johan Högberg

**Affiliations:** Institute of Environmental Medicine, Karolinska Institutet, Box 210, SE-17177 Stockholm, Sweden

**Keywords:** autotaxin (ATX), lysophosphatidic acid (LPA), crystalline silica particles (CSi), endonuclease G (EndoG), mitochondrial outer membrane permeabilization (MOMP), DNA damage, double strand breaks (DSB), micronuclei (MN)

## Abstract

**Simple Summary:**

Crystalline silica particles (CSi) are carcinogenic; however, their genotoxicity is not well characterized. Indirect evidence suggests that retained particles in peripheral bronchioles activate macrophages, causing chronic ROS production. Using human bronchial epithelia in monocultures, we have recently shown that CSi activate ATX at the plasma membrane and that a complex signaling pathway leads to double strand breaks (DSBs). The DSBs were seen within minutes, also in mice inhaling CSi. A remaining question is how DSBs are induced. Using the same models as previously, we now show that ATX signaling leads to endonuclease G translocation from the mitochondria to the nucleus. It was synchronized with DSB formation, and inhibitors prevented both the translocation and the DSBs, supporting a causal role for endonuclease G. The CSi-induced micronuclei formation indicated genomic instability. Additionally, the endonuclease G mediated genotoxicity, which thus affects the respiratory epithelium directly at very low particle doses, might explain the carcinogenic effects of CSi, perhaps even better than ROS-producing macrophages.

**Abstract:**

Crystalline silica particles (CSi) are an established human carcinogen, but it is not clear how these particles cause necessary mutations. A well-established scenario includes inflammation caused by retained particles in the bronchioles, activated macrophages, and reactive oxygen species (ROS) that cause DNA damage. In previous studies, we showed that CSi in contact with the plasma membrane of human bronchial epithelium induced double strand breaks within minutes. A signaling pathway implicating the ATX-LPA axis, Rac1, NLRP3, and mitochondrial depolarization upstream of DSB formation was delineated. In this paper, we provide in vitro and in vivo evidence that this signaling pathway triggers endonuclease G (EndoG) translocation from the mitochondria to the nucleus. The DNA damage is documented as γH2AX and p53BP1 nuclear foci, strand breaks in the Comet assay, and as micronuclei. In addition, the DNA damage is induced by low doses of CSi that do not induce apoptosis. By inhibiting the ATX-LPA axis or by EndoG knockdown, we prevent EndoG translocation and DSB formation. Our data indicate that CSi in low doses induces DSBs by sub-apoptotic activation of EndoG, adding CSi to a list of carcinogens that may induce mutations via sub-apoptotic and “minority MOMP” effects. This is the first report linking the ATX-LPA axis to this type of carcinogenic effect.

## 1. Introduction

The crystallin silica (CSi) particles are an established risk factor for lung cancer, but a remaining uncertainty is how Csi causes necessary mutations [1]. In risk assessments of Csi, it is often suggested that Csi genotoxicity involves so-called indirect effects on epithelial cells via activated inflammatory cells, including ROS-producing macrophages [1,2]. This is a chronic effect maintained by retained particles in distant bronchioles. The indirect support for this mode of action (MoA) is that unrealistically high doses of CSi are needed to induce genotoxicity in epithelial cell models lacking inflammatory cells and that CSi have not been observed to enter nuclei or mitochondria in vivo [2].

In previous studies [3,4,5], we found that CSi in contact with the plasma membrane rapidly induced DNA damage in respiratory epithelial cells in culture and in mice in situ. We documented activation of the DNA damage response (DDR) and double strand breaks (DSBs) within a few minutes and earlier than we detected particle uptake [3,4,5]. In addition, low doses of CSi were effective, and the fast onset effect differed from those previously reported for CSi genotoxicity [2]. We also delineated a signaling pathway from the plasma membrane leading to DSBs. It involves purinergic receptors, ATX, LPA, and Rac1 [3,4]. The downstream events included the phosphorylation of NLRP3 (Ser198) and its colocalization with the mitochondrial outer membrane and mitochondrial depolarization. Inhibitors of ATX, LPA receptors, mitochondrial depolarization, JNK1, and NLRP3 prevented the rapid genotoxicity [3,4,5]. We reasoned that further characterization of this type of DNA damage might be of interest for understanding the carcinogenic effects of CSi.

We also showed that this rapid DNA damage preceded detectable increases in ROS production [3,4], and a remaining question is how these DSBs arise. In this study, we investigate the possibility that a nuclear translocation of mitochondrial endonuclease G (EndoG) may explain the rapid DSB formation. Our hypothesis is based on studies of limited mitochondrial permeability (minority mitochondrial outer membrane permeabilization, MOMP) and sub-lethal apoptosis [6,7,8]. This recently discovered cellular effect can be induced by various stressors, including established carcinogens, and in surviving cells, EndoG may translocate from the mitochondria to the nucleus and cause DSBs, micronuclei (MN), and genomic instability [6,8,9]. In addition, the caspase-activated DNAse (CAD) also has a role in this process [7,9]. In cancer cells, a similar mechanism may operate spontaneously [9]. The sub-lethal apoptosis hypothesis is attractive to us as it may drive cancer development [6,7,8,10]. Furthermore, MN, even in epithelial cells, is used as a biomarker of CSi exposure at workplaces [11,12,13], so studies of MN formation in our models, might give ways to relate our studies to human exposure scenarios. 

Furthermore, we show that low doses of CSi rapidly increase nuclear levels of EndoG in vitro and in vivo and that this effect is mediated via the same signaling pathway as we described previously for DNA damage [3,4], i.e., we indicate a key role for ATX, LPA, and LPA receptors in genotoxicity mediated by EndoG.

## 2. Materials and Methods

### 2.1. Cell Culture 

The 16HBE14o- (16HBE), human bronchial epithelial cells transformed with SV40 large T-antigen, were provided by Prof. Dieter C. Gruenert (University of California, San Francisco, CA, USA). The cells were cultured in EMEM (Bio Whittaker, Lonza) supplemented with 10% (*v*/*v*) inactivated fetal bovine serum (FBS) (Gibco, Thermo Fisher Scientific, Waltham, MA, USA), 100 U/mL penicillin and 100 μg/mL streptomycin mixture (Gibco, Fisher Scientific, USA), and 2 mM L-glutamine (Gibco, Thermo Fisher Scientific, Waltham, MA, USA).

### 2.2. Particles and Reagents

The crystalline silica particles (Min-U-Sil 5) were purchased from the U.S. SILICA Company. The nominal diameter was 1.6 μm. Particle characterization and preparation were performed as described previously [5]. Antibodies against p53BP1 (2675S), γH2AX Ser139 (2577 L), and GAPDH (5174P) were from Cell Signaling Technology, Inc., Beverly, MA, USA. EndoG (sc-365359) was from Santa Cruz (Santa Cruz, CA, USA). EndoG (SAB3500213) was from Sigma-Aldrich (Merck, St. Louis, MO, USA). Inhibitors PF8380 (SML0715), Ki16425 (SML0971), KN62 (12142), MCC950, mitoTEMPO, Cyclosporin A (CsA), and Caspase 3 inhibitors (235420) were purchased from Sigma-Aldrich (Sigma-Aldrich, St. Louis, MO, USA). 

### 2.3. Animal Treatment

The male C57BL/6 mice (8–12 weeks old) were purchased from Charles River (Sulzfeld, Germany). Mice were treated as described previously [3]. In brief, mice were lightly anesthetized with isoflurane and thereafter exposed to silica by intranasal instillation (25 µg silica suspended in 25 µL PBS). Control mice were instilled with PBS. Thereafter, the animals were deeply anesthetized and sacrificed at the times indicated. Lung tissues were collected and fixed in 4% formaldehyde. The experiments involving animals were conducted according to Swedish government norms. The authors have complied with all relevant ethical regulations for animal testing and research. This study was approved by ethical permit N55/15 from the Stockholm ethics committee. 

### 2.4. RNA Interference

The interference RNA (siRNA) against Endonuclease G (s708) was purchased from Invitrogen (Invitrogen, Carlsbad, CA, USA), and the control scramble siRNA was purchased from Santa Cruz Biotechnology (Santa Cruz, CA, USA). 16HBE cells were transfected with siRNA endonuclease G using Lipofectamine RNAiMAX (Thermo Fisher Scientific, Waltham, MA, USA) for 48 h.

### 2.5. Nuclear Extraction

After silica exposure, the whole cell lysate was fractionated with a 0.25 M sucrose buffer. The nuclear lysate was centrifuged at 6000× *g* for 10 min, followed by washing in PBS, and thereafter the nuclear pellet was suspended in 0.25 M sucrose buffer and 2X IPS 2 lysis buffer. The samples were analyzed by Western blotting. 

### 2.6. TUNEL Assay

TUNEL (transferase-mediated deoxyuridine triphosphate-biotin nick end labeling) analysis was performed with a commercially available kit for detecting end-labeled DNA following the manufacturer’s protocol (Sigma, in situ cell death POD, #54421700) and analyzed by confocal microscopy [14].

### 2.7. Comet Assay

The cells were embedded in 0.75% agarose gel and incubated in lysis buffer for 1 h. Alkaline electrophoresis (300 mM NAOH, 1 mM EDTA, pH > 13) was performed for 30 min at 25 V and followed by staining with SYBR Green. The percentage DNA tail was measured with Comet Assay IV (Instem, Stone, UK) software and fluorescent microscopy (Leica, Wien, Austria). 

### 2.8. Immunocytochemistry

The cells were fixed with 4% formaldehyde and permeabilized with 0.1% Triton X-100 in PBS. The cells were stained with the primary antibodies EndoG (sc-365359) and p53BP1 (Cell Signaling Technology, Inc., Beverly, MA, USA). After washing, the cells were incubated with Alexa 594 secondary antibodies (Thermo Fisher Scientific, Waltham, MA, USA). Images were acquired by using a 63x oil immersion objective on a Zeiss LSM 900 Airy confocal laser scanner microscope (Zeiss, Göttingen, Germany). A minimum of 50 cells were counted for each sample. 

### 2.9. Immunohistochemistry

The mouse lung tissue was fixed and prepared as paraffin-embedded sections. The slides were deparaffinized and rehydrated, followed by antigen retrieval using citrate buffer in a microwave (650 W for 20 min). The slides were stained with primary antibodies EndoG (SAB3500213), γH2AX (JBW301, Merck), or p53BP1 (Cell Signaling), and DAPI (Life Technologies). Images were acquired by using a Zeiss LSM 900 Airy confocal laser scanner microscope (Zeiss, Göttingen, Germany). The 3D view of Z-stack images was measured in Imaris image analysis software (Bitplane AG, Zurich, Switzerland). The quantification of signal intensity was analyzed by ImageJ software. A minimum of 50 cells per condition were quantified. 

### 2.10. Western Blotting

The western blotting was performed as described previously [3]. Membranes were probed with primary antibodies against EndoG (sc-365359), GAPDH (5174P), and γH2AX Ser139 (2577 L). Densitometric analysis was performed using Image J software. 

### 2.11. Statistical Analysis

All experiments were performed at least in triplicate. Statistical analysis and graphs were performed in GraphPad Prism (version 9.0 of GraphPad software, Inc., San Diego, CA, USA). The data on Western blotting are presented as means ± SD; statistical differences were assessed by ANOVA with Bonferroni’s post hoc test (significance rated as *p* < 0.05). The data on Comet assay, immunocytochemistry, and immunohistochemistry are presented as medians with a 95% confidence interval; statistical differences were measured by ANOVA with the Kruskal-Wallis test (significance rated as *p* < 0.05). 

## 3. Results

### 3.1. Silica-Induced Translocation of EndoG in Respiratory Epithelial Cells In Vitro

**Figure 1:**
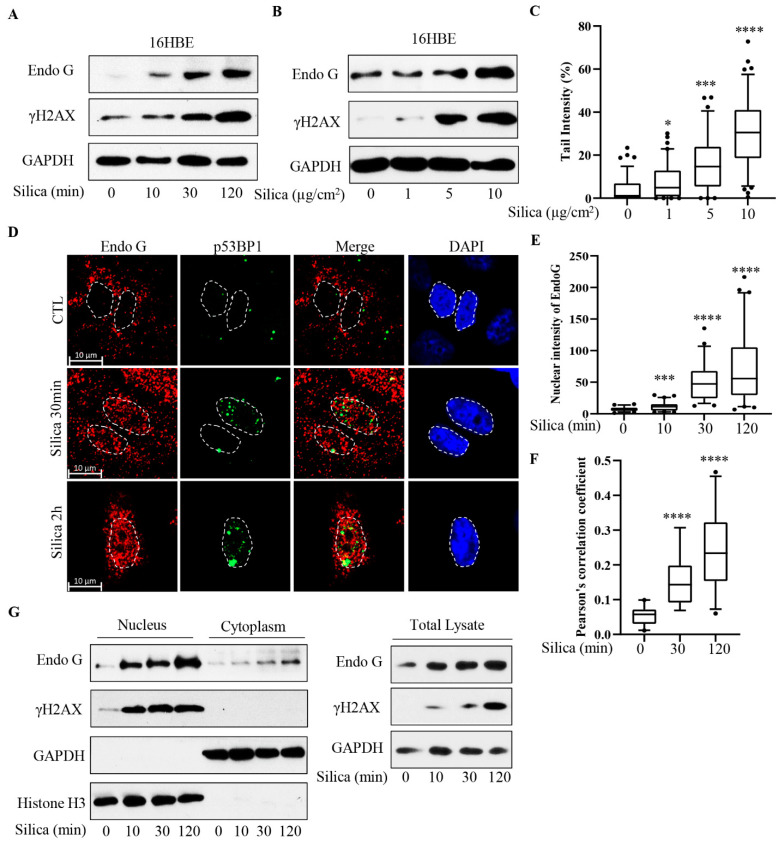
Silica-induced translocation of EndoG in respiratory epithelial cells in vitro. In (**A**), 16HBE cells were exposed to silica (5 µg/cm^2^) for times indicated. Cell lysates were analyzed for γH2AX and EndoG by Western blotting. In (**B**,**C**), 16HBE cells were exposed to silica (1, 5, 10 µg/cm^2^) for 2 h. EndoG and γH2AX were analyzed by Western blotting (**B**) and DNA damage was analyzed by Comet assay (**C**). In (**D**), representative confocal images showing EndoG (red), p53BP1 (green) and nuclear (blue) staining in 16HBE cells exposed to silica (5 µg/cm^2^) for 30 min and 2 h. In (**E**), nuclear intensity of EndoG was quantified by using ImageJ software. In (**F**) EndoG and p53BP1 colocalization was quantified by Pearson’s correlation coefficient using Imaris software. In (**G**), cells were exposed to silica (5 µg/cm^2^) for times indicated and then subjected to nuclear extraction. EndoG and γH2AX levels in nucleus were analyzed by Western blotting. Bars show median with 95% confidence interval. All experiments were performed at least in triplicate. * *p* < 0.05, *** *p* < 0.001, **** *p* < 0.0001, compared to control (CTL) as determined by ANOVA.

Figure 1 shows that CSi (5 µg/cm^2^) rapidly induces γH2AX, a marker of DNA damage and DSBs, in human respiratory epithelial cells (16HBE) in culture. Previously, we showed increased levels of γH2AX at 5 min and later [3]. Here, we incubated cells for 10–120 min and show that γH2AX levels increased with time. It was also observed that the total cellular amount of detectable EndoG increased with time (Figure 1A, Supplementary Figure S1). A similar increase has been shown, e.g., after IR exposure [8]. This EndoG induction was so fast that we anticipate that the increase might reflect the release of already synthesized proteins, e.g., chaperone proteins [15]. In Figure 1B (Supplementary Figure S1), a dose relationship is shown, and CSi at 1 µg/cm^2^ also had effects, even though it is unclear if it affected EndoG levels. Figure 1C shows a Comet assay analysis of increasing doses, thus confirming DNA damage and strand breaks. The data on DNA damage are in line with our previous data [3,4].

In Figure 1D, confocal images are presented. Under control conditions, the staining for EndoG was localized to the cytoplasm. Hardly any nuclear staining was detected. EndoG translocation to the nucleus has been associated with apoptosis [16], and we used the topoisomerase inhibitor and potent apoptosis inducer, camptothecin [3], as a positive control for EndoG translocation. Within 2 h camptothecin translocated EndoG to the nucleus (not shown). 30 min after CSi addition, the cytoplasmic spots were less intensely stained, and the nucleus contained spots with EndoG staining. Additionally, it is also shown as a graphical presentation of confocal data. A significant increase of nuclear EndoG-positive staining was seen at 10 min and increased further with time (Figure 1E). These alterations indicate EndoG translocation to the nucleus in response to CSi. 

We also stained for phosphorylated 53BP1 (p53BP1), another marker for DNA damage and DNA damage response (DDR) [17]. This marker was increased at 30 and 120 min in the nucleus (Figure 1D), further indicating an accumulation of DSBs in the nucleus and supporting the data on γH2AX (Figure 1A,B). We also analyzed p53BP1—EndoG co-localization, seen as yellow spots in Figure 1D. These dots increased time-dependently (Figure 1F) and were in line with data on γH2AX (Figure 1A). This indicates a correlation between EndoG translocation and DSB formation. The p53BP1 data are consistent with data showing that IR induces p53BP1 dots in the nucleus already after 5 min [18]. Our data thus indicate that EndoG foci in the nucleus harbor DSBs. Similar dots stained for γH2AX and EndoG were documented 10 days after IR exposure [8].

In order to further confirm a translocation of EndoG to the nucleus, we performed cell fractionation (Figure 1G, Supplementary Figure S1). The data showed that there was a time-dependent increase of EndoG in the nuclear fraction that was paralleled by increasing levels of γH2AX in the same fraction. In the cytoplasm and in the total lysate, we detected increasing levels of EndoG (Figure 1G), as in Figure 1A,B. These data confirm a CSi-induced rapid translocation of EndoG to the nucleus in cell cultures.

### 3.2. Translocation of EndoG In Vivo

**Figure 2:**
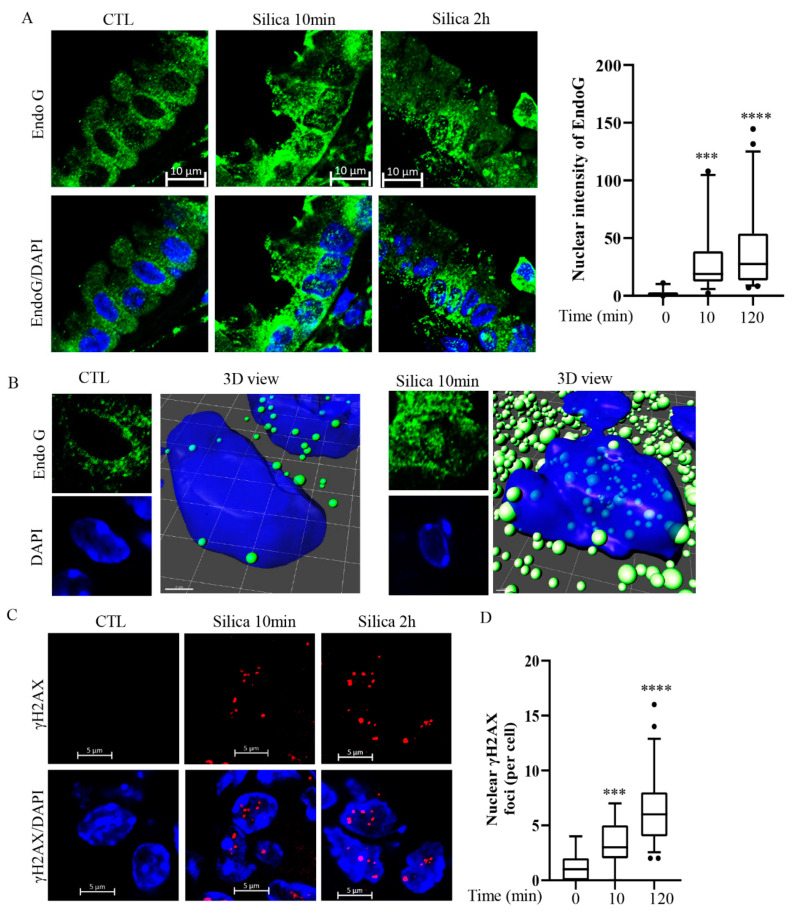
Silica-induced nuclear translocation of EndoG in mouse lung in vivo. Mice were exposed to a single dose of silica (25 µg/mouse) or to PBS as control (CTL) by nasal instillation. In (**A**), representative confocal images showing EndoG (green) and nuclear (blue) staining in mouse lung sections. Nuclear EndoG was quantified by ImageJ software. In (**B**), images from A using Z-stack were analyzed as 3D view by Imaris software. In (**C**) representative confocal images showing γH2AX (red) and nuclear (blue) staining in mouse lung sections. Nuclear γH2AX foci was quantified by ImageJ software (**D**). Bars show median with 95% confidence interval. All experiments were performed at least in triplicate. *** *p* < 0.001, **** *p* < 0.0001 compared to PBS, as determined by ANOVA.

The lung tissues from mice were analyzed 10 or 120 min after exposure to CSi via nasal inhalation (25 µg in PBS/mouse) [3,4]. We employed confocal microscopy and used antibodies against EndoG and γH2AX. The data in Figure 2A show that already after 10 min dots stained for EndoG can be seen in nuclei in situ, whereas no dots were detected under control conditions. The dots became more prominent with time, as shown in the graph. Figure 2B shows a 3D representation of a nucleus in situ stained for EndoG. Under control conditions, a limited number of EndoG dots were seen outside the nucleus, and some of them appeared to be localized above the nucleus. After 10 min CSi exposure, several dots were detected in the nucleus. This is further support for the localization of EndoG to the nucleus in response to CSi. Figure 2C shows lung slides stained for γH2AX and Figure 2D shows a graphical presentation. Several intranuclear γH2AX foci can be seen at 10 min and more at 120 min. This indicates that CSi translocate EndoG both in vitro and in vivo, and with a similar time scale.

### 3.3. Inhibition of EndoG Prevents DNA Damage and CSi Does Not Induce Apoptosis

**Figure 3:**
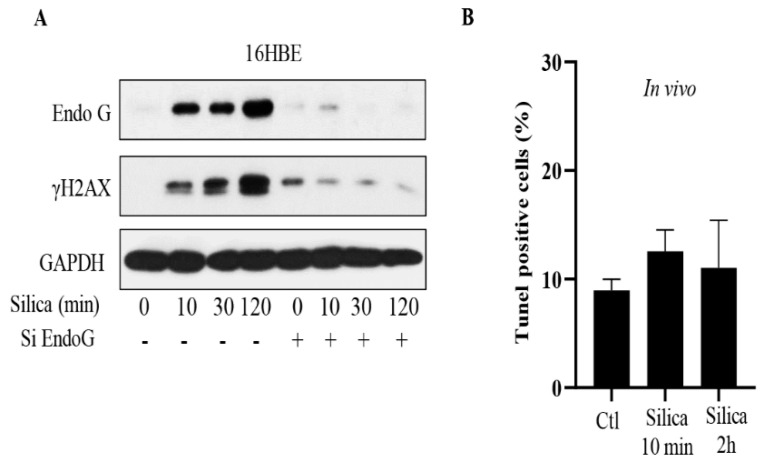
Inhibition of EndoG prevents silica-induced DNA damage. In (**A**), 16HBE cells were transfected with siRNA EndoG (30 pM) for 24 h, and then exposed to silica (5 µg/cm^2^) for times indicated. Cell lysates were analyzed for EndoG and γH2AX by Western blotting. In (**B**), TUNEL assay was performed to assess apoptotic cells in mouse lung tissue. Quantification of TUNEL positive cells was analyzed by Image J software. Bars show means ± SD. All experiments were performed at least in triplicate.

The data presented in Figure 1D,E indicate a correlation between nuclear EndoG levels and the two DNA damage markers,γH2AX and p53BP1. To further investigate the role of EndoG in DSB formation, we also tested siRNA for EndoG. As shown in Figure 3A, we found that after pretreatment of the cells with siRNA, EndoG levels were undetectable even after CSi exposure. The finding that γH2AX remained at control levels indicates that EndoG caused DSB formation (Figure 3A, Supplementary Figure S2). These data are in line with the effects of EndoG inhibitors or genetic inactivation of EndoG enzyme activity on starvation- or etoposide-induced DNA damage [19].

In our previous studies, we found no signs of increased apoptosis with CSi exposure (5 µg/cm^2^) within 24 h [3,4,5]. The data presented in Figure 3B is in line with our previous studies. As can be seen, the fraction of TUNEL-positive cells was not markedly increased by CSi exposure for 2 h, whereas a positive control, camptothecin, caused significant cell death (not shown). A similar lack of effect was observed in vivo (Figure 3B). These data, taken together with the information that we used lower doses of CSi than used in most in vitro studies of CSi [2], indicate that EndoG was translocated to the nucleus without causing extensive cell death. This conclusion is further supported by the observation that the dose we used in our mouse experiments was 100 times lower than the dose (inhalation of 2.5 mg silica/mouse) used in a three-month study, of mice [20]. However, in that study, more TUNEL-positive cells were observed three months after the single inhalation [20]. 

### 3.4. Inhibition of ATX Inhibits the EndoG Translocation and DNA Damage

**Figure 4:**
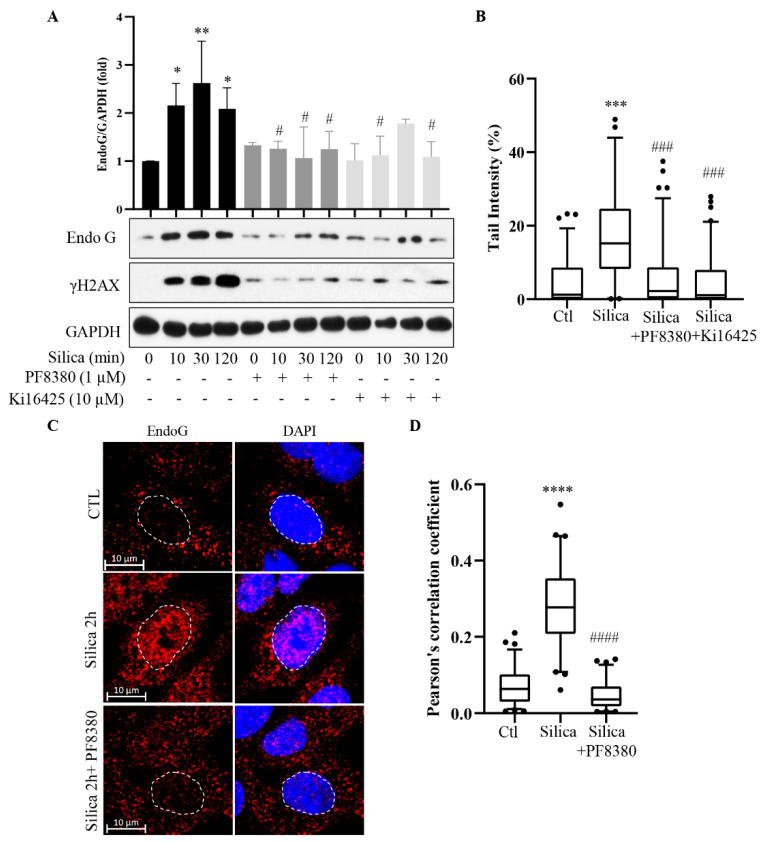
ATX inhibition reduces silica-induced EndoG translocation to nucleus and DNA damage. 16HBE cells pretreated with the ATX inhibitor PF8380 (1 µM) or the LPA inhibitor Ki16425 (10 µM) for 1 h, and then exposed to silica (5 µg/cm^2^) for times indicated. In (**A**), cell lysates were analyzed for EndoG and γH2AX by Western blotting. In (**B**), cells from A were analyzed for DNA damage using Comet assay. In (**C**), representative confocal images of 16HBE cells with EndoG (red) and nuclear (blue) staining. Controls (CTL) and cells exposed to silica for 2 h +/− PF8380 are shown. In (**D**), EndoG in DAPI stained areas is quantified. Bars in A show means ± SD. Bars in (**B**,**D**) show median with 95% confidence interval. All experiments were performed at least in triplicate. * *p* < 0.05, ** *p* < 0.01, *** *p* < 0.001, **** *p* < 0.0001 compared to control. # *p* < 0.05, ### *p* < 0.001, #### *p* < 0.0001 compared to silica exposure, as determined by ANOVA.

In previous studies, we delineated a signaling pathway, partially by using different small molecular chemical inhibitors that prevented DNA damage [3,4,5]. Here, we investigated if a set of these inhibitors also affected EndoG translocation. First, we used PF8360 (an ATX inhibitor) and Ki16425 (an inhibitor of LPA receptors 1 and 3) (Figure 4A, Supplementary Figure S2). Both of these chemicals inhibited the CSi-induced increase in EndoG levels as well as the increase in γH2AX levels. They also inhibited the DNA damage detected by the Comet-assay (Figure 4B). Figure 4C shows confocal images and that PF8380 inhibited the EndoG translocation to the nucleus. Figure 4D shows colocalization between EndoG staining and DAPI staining. 

### 3.5. Inhibition of Mitochondrial Depolarization Prevents Mitochondrial Depletion of EndoG and Its Nuclear Translocation

**Figure 5:**
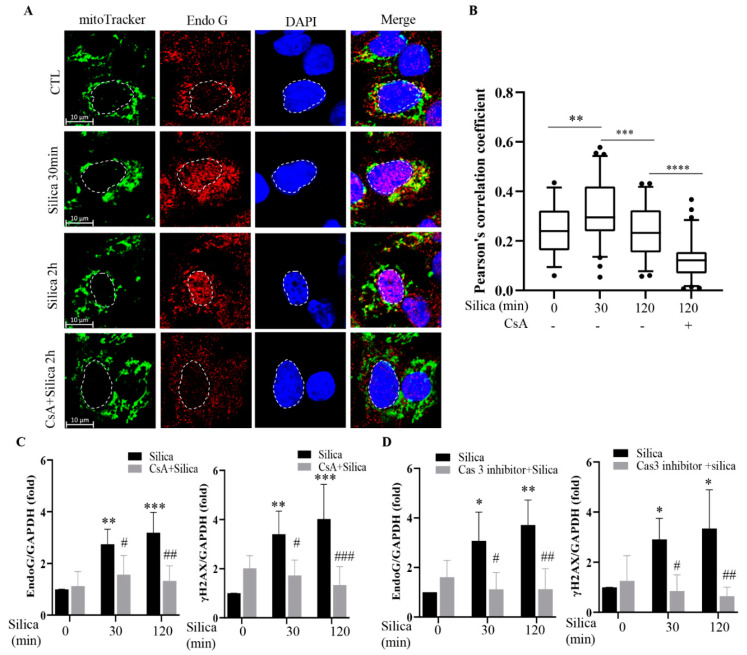
Cyclosporin A (CsA) prevents silica-induced depletion of mitochondrial EndoG, EndoG translocation and DNA damage. In (**A**), representative confocal images showing EndoG (red), mitoTracker (green), and nuclear (blue) staining in 16HBE cells exposed to silica (5 µg/cm^2^) for times indicated. Cells pretreated with CsA (20 µM) for 1 h are shown in the bottom row. In (**B**), EndoG and mitoTracker colocalization is quantified. In (**C**) 16HBE cells were pretreated with CsA (20 µM) for 1 h and then exposed to silica (5 µg/cm^2^) for times indicated. Cell lysates were analyzed for EndoG and γH2AX by Western blotting. In (**D**) 16HBE cells pretreated with Caspase-3 inhibitor (10 µM) for 1 h and followed by silica (5 µg/cm^2^) for times indicated. Cell lysates were analyzed for EndoG and γH2AX by Western blotting. Bars in B show median with 95% confidence interval. Bars in C and D show means ± SD. All experiments were performed at least in triplicate. * *p* < 0.05, ** *p* < 0.01, *** *p* < 0.001, **** *p* < 0.001 compared to control. # *p* < 0.05, ## *p* < 0.01, ### *p* < 0.001 compared to silica exposure, as determined by ANOVA.

EndoG is a mitochondrial protein under unstressed conditions [16]. In Figure 5A, we show confocal images of cells double-stained for EndoG and the mitochondrial marker mitoTracker. They are like those shown in [21] and confirm a mitochondrial localization of EndoG under control conditions. Figure 5A also shows a translocation of EndoG to the nucleus after exposure to CSi for 30 or 120 min, as shown above. Furthermore, the EndoG staining and mitoTracker staining gave yellow dots in the cytoplasm when merged, indicating close colocalization in mitochondria. This indicates that at 30 min substantial amounts of EndoG remained in the mitochondria, which we relate to the increasing total EndoG levels (as seen, e.g., in Figure 1A). At 120 min the yellow staining was weakened (Figure 5A,B), indicating a depletion of mitochondrial EndoG. 

The mitochondrial depolarization plays a critical role in sub-lethal apoptosis signaling [6,7,8]. In our previous studies [3,4], we stabilized mitochondria with cyclosporin A (CsA) to prevent DNA damage. The CsA blocks the mitochondrial permeability transition pore (MPTP) and prevents depolarization [22]. Here, we used CsA to investigate the role of depolarization in EndoG translocation. As shown in Figure 5A,B, CsA pretreatment for 120 min prevented the effect of CSi on EndoG translocation as well as the merged, yellow staining in the cytoplasm (Figure 5A,B). This indicates a key role for mitochondrial depolarization in the EndoG translocation. An inhibiting effect of CsA is also demonstrated by the analysis of EndoG in cell lysates (Figure 5C, Supplementary Figure S3). The effects of CsA shown in Figure 5A–C confirm a connection between mitochondrial depletion and nuclear accumulation of EndoG. As expected from previous studies [3,4], CsA also prevented the increase in γH2AX levels (Figure 5C). Caspase-3 is implicated in sub-lethal apoptosis signaling and DNA damage [23,24]. In line with the data presented in [8], we found that a Caspase-3 inhibitor prevented EndoG induction as well as the increase in γH2AX levels (Figure 5D, Supplementary Figure S3).

### 3.6. Common Signaling Pathway for EndoG Translocation and DNA Damage

**Figure 6:**
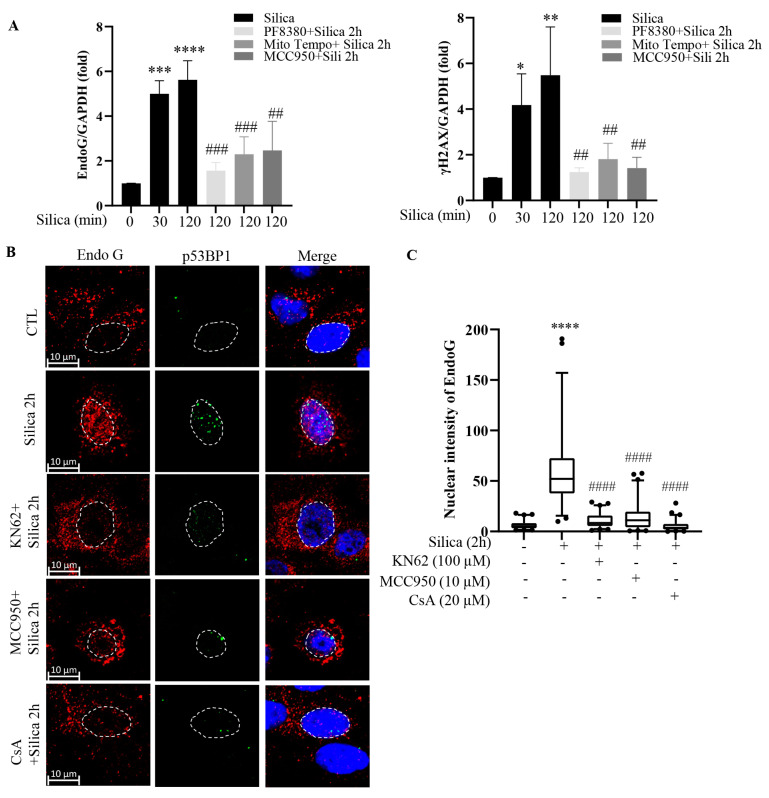
Silica-induced EndoG translocation and DNA damage are caused by the same signaling pathway. In (**A**), 16HBE cells were pretreated with PF8380 (1 µM), mitoTempto (1 µM), or MCC950 (10 µM) for 1 h and then exposed to silica (5 µg/cm^2^) for times indicated. Cell lysates were analyzed for EndoG and γH2AX by Western blotting. Densitometric analysis of three different experiments is shown. (**B**) Representative confocal images showing EndoG (red), p53BP1 (green), and nucleus (blue) staining in 16HBE cells pretreated with KN62, MCC950, and CsA and followed by silica (5 µg/cm^2^) exposure for 2 h. (**C**) Nuclear EndoG was quantified by ImageJ software. Bars in (**A**) show means ± SD. Bars in (**C**) show median with 95% confidence interval. All experiments were performed at least in triplicate. * *p* < 0.05, ** *p* < 0.01, *** *p* < 0.001, **** *p* < 0.0001 compared to control. ## *p* < 0.01, ### *p* < 0.001, #### *p* < 0.0001 compared to silica exposure, as determined by ANOVA.

Next, we tested three additional chemical inhibitors previously found to prevent CSi-induced DNA damage [3,4,5]. In line with the data shown above, EndoG and γH2AX levels were increased by CSi at 30 min and at 2 h, and PF8380 prevented both effects (Figure 6A, Supplementary Figure S3). Similarly, MCC950 (a NLRP3 inhibitor) and mitoTEMPO (a mitochondrial antioxidant) [3,4] inhibited both responses (Figure 6A). In Figure 6B, we used p53BP1 as an indicator of DSBs. Apart from CsA, we pretreated cells with KN62 (an unspecific P2X7 inhibitor) and MCC950 (the NLRP3 inhibitor). All three compounds inhibited the translocation of EndoG to the nucleus as well as the p53BP1 response (Figure 6B). Figure 6C is a graphical presentation of the confocal data. Taken together, the data presented in Figure 4 and Figure 5 show that the CSi-induced EndoG translocation to the nucleus is inhibited by a set of chemical inhibitors selected for their ability to prevent DSB formation. As discussed previously [3,5], these chemicals inhibit different steps in a siganling pathway leading to DNA damage, and data shown here indicate that a common siganling pathway triggers EndoG translocation and DSB formation. Taken together with the siRNA-EndoG data in Figure 3A, they indicate a direct role for EndoG in DSB formation. 

### 3.7. CSi-Induced Micronuclei Formation

**Figure 7:**
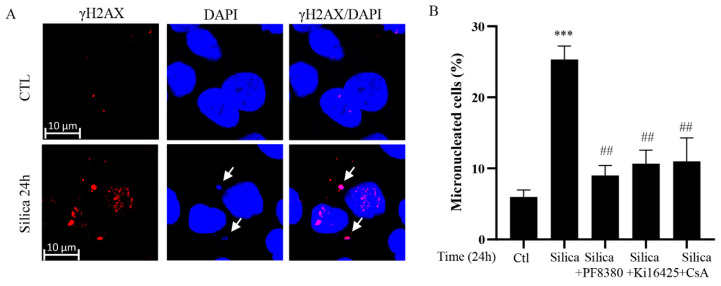
Silica-induced micronuclei formation in 16HBE cells. In (**A**), representative confocal images showing γH2AX (red) and nuclear (blue) staining in 16HBE cells exposed to silica (5 µg/cm^2^) for 24 h. (**B**) 16HBE cells pretreated with PF8380, Ki16425, or CsA for 1 h and then exposed to silica (5 µg/cm^2^) for 24 h. The number of micro-nucleated cells (%) was analyzed employing confocal microscopy. Bars show means ± SD. All experiments were performed at least in triplicate. *** *p* < 0.001 compared to control. ## *p* < 0.01 compared to silica exposure, as determined by ANOVA.

The EndoG translocation to the nucleus has previously been associated with micronuclei formation [6,8], and we cultured CSi-exposed 16HBE cells for 24 h. As can be seen in Figure 7A, this resulted in the appearance of dots stained for DAPI in the cytoplasm, representing micronuclei. These dots were also co-stained for γH2AX, indicating unrepaired DNA remaining in the micronuclei. In addition, pretreatment with ATX and LPA receptor inhibitors and the mitochondrial stabilizer CsA reduced micronuclei formation, as presented in the graph (Figure 7B). This is in line with previous studies on sub-lethal apoptosis [6,7,8], and suggests that cells entered the cell cycle with unrepaired DSBs. This indicates that CSi have the capacity to cause a type of DNA damage that has been associated with several other well-known carcinogens [6,7,8]. 

## 4. Discussion

Several studies have demonstrated the carcinogenic effects of EndoG and/or CAD in connection with sub-lethal apoptotic signaling [6,7,8,9,10,25,26,27]. The data presented here indicate a role for EndoG in CSi-induced DNA damage. In the in vitro and in vivo experiments, it is observed that EndoG is translocated to the nucleus within minutes after CSi exposure, that the timing is consistent with a role in DSB formation, and that EndoG knockdown prevents DSBs. In addition, we also show that inhibitors of CSi-induced rapid DSB formation also inhibit EndoG translocation. To our knowledge, this is the first report connecting the ATX-LPA axis to EndoG nuclear translocation and ensuing DSBs. Our data are important for a more comprehensive understanding of CSi-induced genotoxicity. Further, they indicate a mode of action (MoA) for CSi genotoxicity that deviates from previously suggested MoAs invoking inflammatory cells and ROS-induced DNA damage [2]. 

A key factor is partial mitochondrial depolarization, which triggers minority MOMP [7,10]. We showed here and previously [3,4] that the ATX-LPA axis plays a role in mitochondrial depolarization. Such a capability of the ATX-LPA axis is supported by other studies [28,29]. They showed that LPA levels were increased in brain tissue in vivo and in vitro in response to hypoxia, that LPA induced mitochondrial impairment and endothelial permeability, and that ATX and LPA inhibitors counteracted mitochondrial dysfunction [28,29]. Together with our data, these studies indicate that ATX—mitochondrial stress signaling is important for cell adaptations to different stressors. Another comment relating to the ATX-LPA axis is that it has been shown that EndoG translocation and subsequent DSB formation activate ATM (pATM) and that pATM plays an important role for sustaining growth and stemness following spontaneous EndoG nuclear activation [9]. In line with this, we previously showed increasing levels of pATM after CSi exposure and that ATM knockdown prevented an increase in ATX levels [5]. It is thus possible that pATM sustains or amplifies ATX signaling in a complex interplay at the cell surface.

The role of ROS is another key question for CSi-induced carcinogenicity. Thus, in the traditional MoA scenarios for CSi genotoxicity, ROS attack DNA [1,2], whereas we here provide evidence for EndoG-induced DSBs. In line with others [9], we see EndoG as an alternative to ROS, and in previous papers, we presented evidence against ROS acting as the DNA damaging agent [3,4]. However, minor ROS alterations have a role in the ATX-LPA dependent signaling of EndoG translocation, and this dependence on ROS can fully explain the inhibiting effect of the antioxidant mitoTEMPO shown here and previously [4]. Another question is whether CAD acted in parallel with EndoG in CSi-induced DSB formation. Thus, it has been shown that some stressors activate CAD [7,9,24,30,31], and we did not properly address this possibility. We observed a strong inhibiting effect in our siRNA experiments (Figure 3A), but the other inhibitors might have affected both EndoG and CAD, so additional studies are needed to exclude a role for CAD. 

We did not detect signs of cell death in our experiments. This is consistent with the fact that we used lower doses [3,4,5] than were previously used in cell tests for genotoxicity [2] and in in vivo experiments [20]. Furthermore, the mouse experiment indicates that the dose level used was well tolerated, even though many, if not the majority, of the bronchial epithelial cells exhibited signs of nuclear EndoG translocation. This argues that EndoG translocation is not necessarily apoptotic, as was assumed in initial studies of EndoG translocations [16]. Consequently, the fate of cells affected by EndoG translocations needs further study. Informative for future studies might be the sub-lethal response in gastric epithelium infected by Helicobacter pylori [31]. The biopsies of infected human gastric epithelium revealed a high proportion of caspase-3-positive cells (used as markers of sub-lethal apoptosis signaling) [31], indicating that many surviving but infected gastric epithelial cells can express sub-lethal apoptosis signals. Of further interest is that the biopsies showed increased pATM levels, indicating DNA damage. This correlated to caspase-3-positivity, which is consistent with a role for sub-lethal apoptosis in the carcinogenic effect of *H. pylori* infection.

The EndoG and/or CAD nuclear translocation in surviving cells has been associated with genomic instability and MN formation [6,7,8,10,26]. Thus, MN formation in our model was not surprising. It is important as it connects our findings to the CSi biomonitoring at workplaces [11,12,13]. Of particular interest is MN in buccal epithelial cells, and that buccal cells exhibited signs of acute toxicity [11,12,13]. This indicates that in humans, MN in buccal cells is caused by the acute effects of CSi, and not by sequestered particles in distant peripheral bronchioles [1,2]. Our mouse data suggest that more proximal bronchial epithelium in humans might be affected in a similar acute way as are buccal cells. Additionally, from this perspective and considering the well-established immunological effects of CSi exposure [32], it is intriguing that it has been suggested that pathogen-induced sub-lethal signaling in epithelial cells might be an important physiological way to activate the immune system [24,30,31]. It seems reasonable to assume that future studies of particles such as CSi might give further support for this view and indicate that epithelial cells in the upper part of the respiratory tree are important for CSi-induced pathology.

We find no previous study connecting particles to sub-lethal apoptosis siganlling. Comparing the effects of CSi and other particles is therefore a matter for future research. Of interest should be the definition of critical dose parameters for non-apoptotic EndoG translocations caused by CSi and other particles. Comparing such data may provide information about their carcinogenic potential. The carcinogens used in previous studies of non-lethal apoptosis (see, e.g., [6,8]) should also be of interest. Previously, we used FCCP and found that this mitochondrial uncoupler induced DNA damage similar to that induced by CSi [3]. If FCCP, similar to CSi, translocates EndoG at significantly lower doses than those that trigger apoptosis, then FCCP should also be studied.

There are non-genital cancer types that are suspected to be more common among men than among women due to intrinsic but uncharacterized biological factors [33,34,35]. However, some of these cancer types have been highlighted in studies of minority MOMP. These include melanoma [10], liver cancer [6], and esophageal cancer [27]. This might be a coincidence; however, EndoG has been ascribed basal male-specific genetic effects. Thus, a recent study indicates a role for EndoG in the degradation of human male mtDNA [36], as has previously been shown for *C. elegans* [19]. In the former study of infertile men and their sperms, a low copy number of the EndoG gene correlated to a frequent occurrence of SNPs in genes coding for the respiratory chain complex [36]. These SNPs relate to increased oxidative stress [37], and oxidative stress is one of these intrinsic biological factors that might explain why men are more often affected by cancer [38]. So, a dysfunctional regulation of EndoG might influence the risk for several cancer types that are more prevalent among men. 

We used lower doses of CSi [3,4,5] than those previously regarded as risk-free in in vitro assays for genericity [2], and our studies might be of interest for defining exposure conditions that do not increase the risk for lung cancer. Establishing such exposure conditions is complex, but a well-characterized MoA for genotoxicity together with the lowest exposure level activating genotoxicity can be guiding factors [39]. We think the data presented here contribute to the establishment of a novel MoA for CSi genotoxicity. By combining human MN and experimental studies, as those described here, we think exposure levels without extra cancer risk should be possible to define with good accuracy.

A limitation of our study is the lack of more long-term experiments showing, e.g., the destiny of cells harboring micronuclei. Although MN are good biomarkers for predicting cancer [13,40], further studies are needed to understand the long-term effects of our findings and their relevance for workplace exposures to CSi. Another related limitation is that we cannot compare the dose we used here (5 µg/cm^2^ cells) to exposure levels reported in epidemiological studies on associations between CSi and cancer in humans. In these studies, the exposure is often reported as levels of CSi in inhaled air (mg/m^3^ air) multiplied by years of exposure. However, further studies using MN as an endpoint in our models might be a way to approach this question, as MN in buccal cells might be acute effects and as workplace exposure levels are given as mg/m^3^ air [11,12,13].

## 5. Conclusions

Our data indicate a novel MoA for CSi-induced genotoxicity. It involves ATX activity and LPA receptors in a complex signaling pathway that starts at the plasma membrane and results in EndoG translocation to the nucleus and ensuing DSB formation. Further studies are needed to understand its role in lung carcinogenesis and whether it raises questions about new preventive strategies for CSi exposure.

## Data Availability

Not applicable.

## Supplementary Materials

The following supporting information can be downloaded at: https://www.mdpi.com/article/10.3390/cancers15030865/s1, Figures S1–S3: Triplicated experiments of Western blot are presented in Figure 1, Figure 3, Figure 4, Figure 5 and Figure 6.
