# Supplementary material for: ATX-LPA-Dependent Nuclear Translocation of Endonuclease G in Respiratory Epithelial Cells: A New Mode Action for DNA Damage Induced by Crystalline Silica Particles
Source: Cancers (Basel). 2023 Jan 30;15(3):865. doi: 10.3390/cancers15030865 (PMC9913843; doi:10.3390/cancers15030865)
Supplement: Supplementary file 1 [file cancers-15-00865-s001.zip › cancers-2074222-supplementary.pdf]

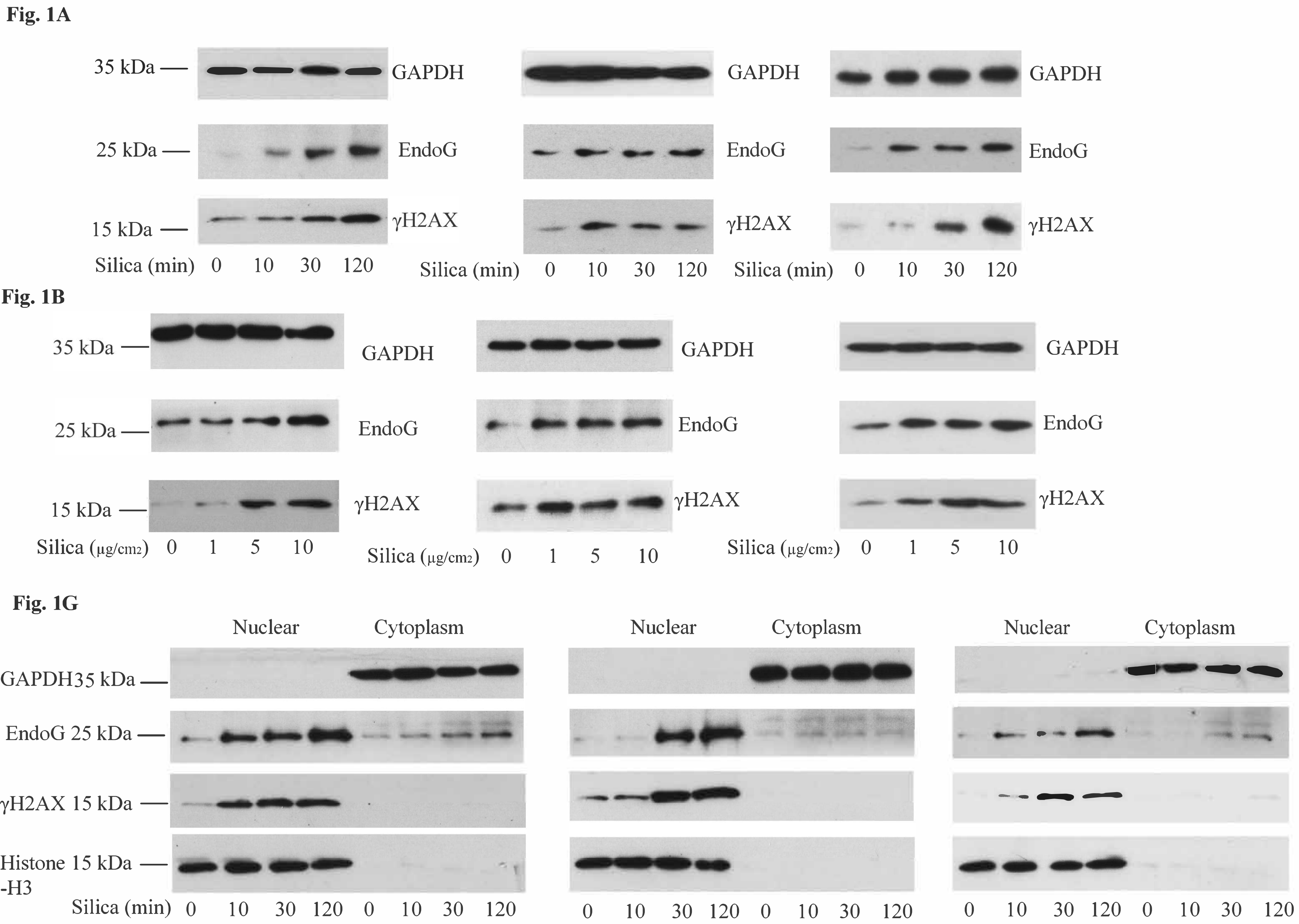

Figure S1: Triplicate experiments of Western blot in Figure 1A, 1B, 1G.

Fig. 3A

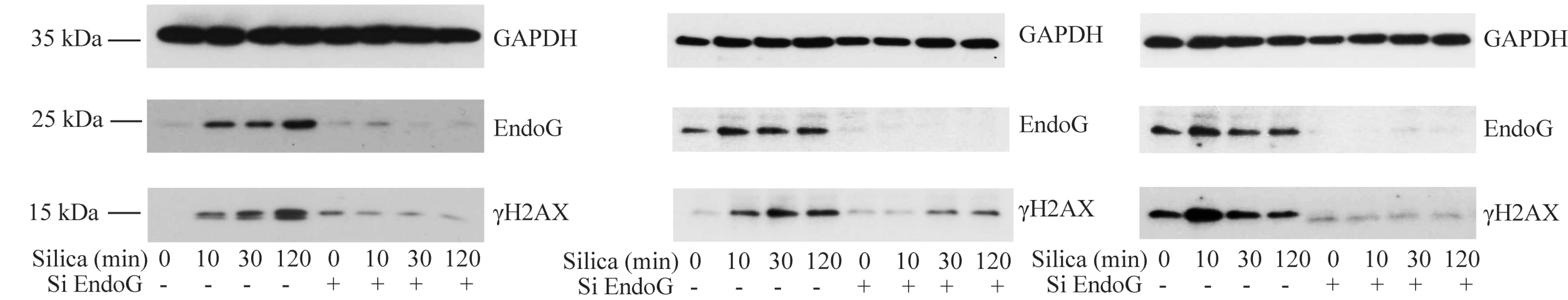

Fig. 4A

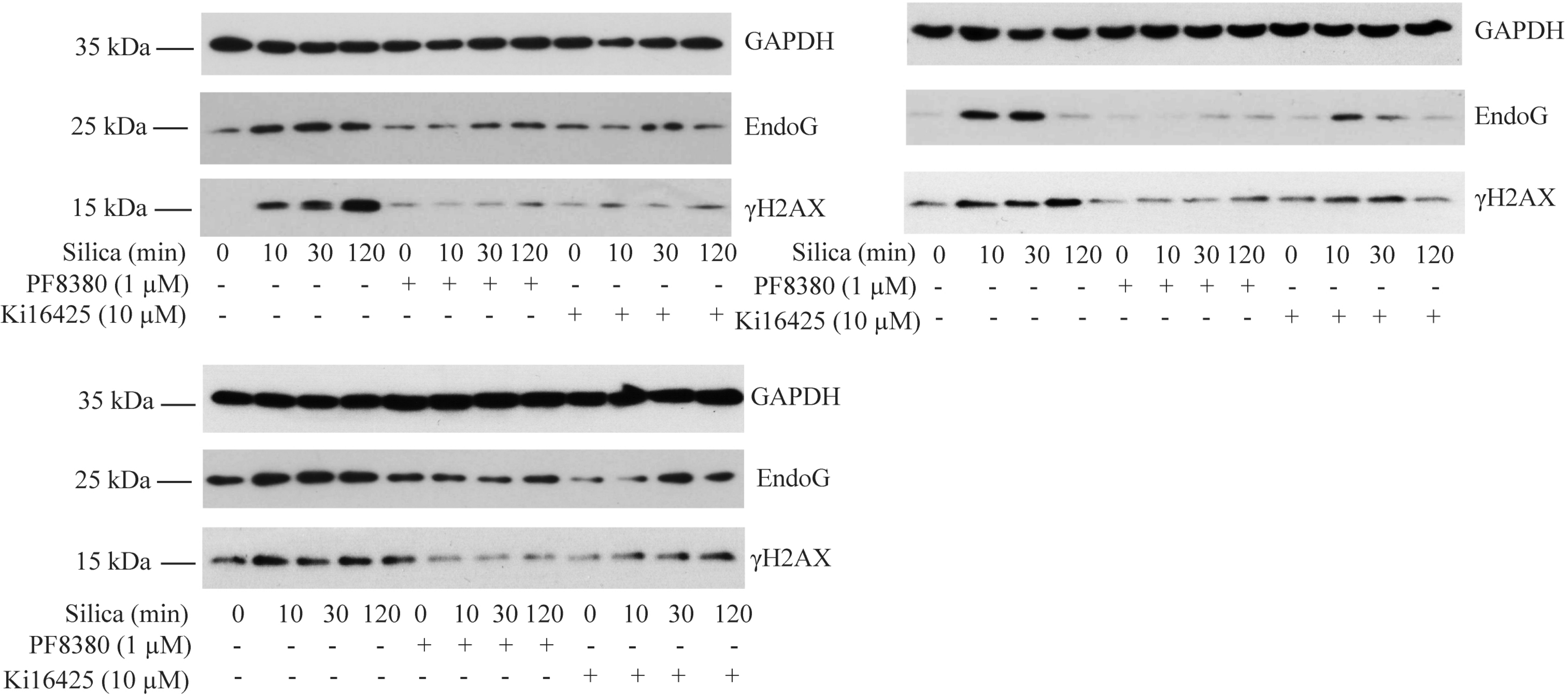

Figure S2: Triplicate experiments of Western blot in Figure 3A, 4A.

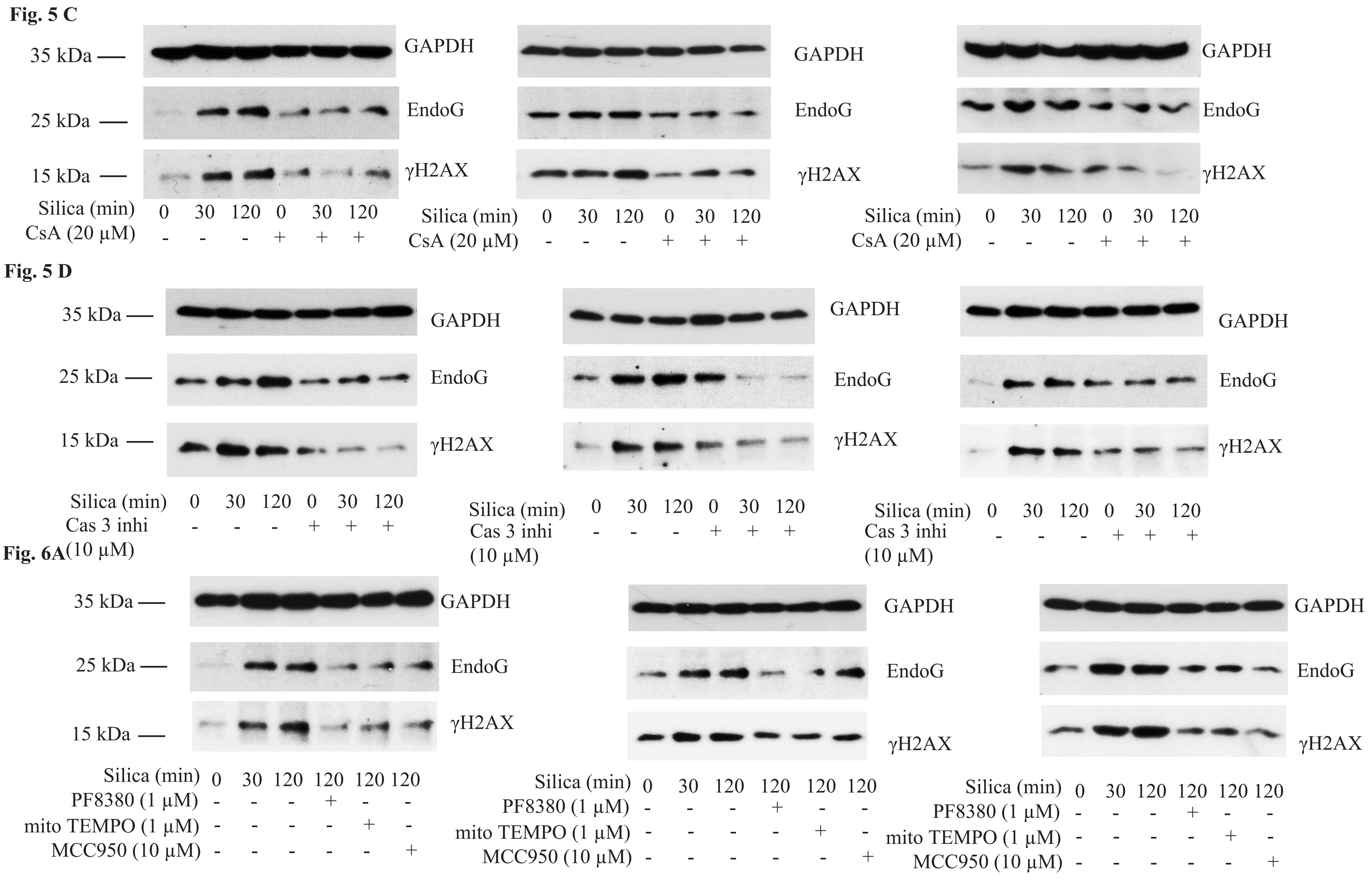

Figure S3: Triplicate experiments of Western blot in Figure 5C, 5D, 6A.
